# Electrophysiological Changes Before and After Glaucoma Filtration Surgery in the Mid-Postoperative Period: A Retrospective Study

**DOI:** 10.7759/cureus.117138

**Published:** 2026-09-29

**Authors:** Ryusuke Hashimoto, Gen Miura, Azusa Yamagishi, Yuta Kitamura, Takayuki Baba

**Affiliations:** 1 Opthalmology, Chiba University, Chiba, JPN; 2 Ophthalmology, Chiba University Hospital, Chiba, JPN; 3 Ophthalmology and Visual Science, Chiba University Graduate School of Medicine, Chiba, JPN; 4 Ophthalmology, Chiba University, Chiba, JPN

**Keywords:** choroidal detachment, electroretinography, glaucoma, glaucoma filtration surgery, reteval

## Abstract

Introduction

Changes in retinal function during the first days after glaucoma filtration surgery have been documented with electroretinography (ERG), but little is known about later time points, particularly in eyes operated on without concurrent cataract surgery and in eyes that develop choroidal detachment (CD). We examined ERG parameters before and after filtration surgery performed alone and explored, as a hypothesis-generating analysis, whether the responses differed between eyes with and without postoperative CD.

Methods

Thirty-two eyes of 32 patients who underwent filtration surgery alone at Chiba University Hospital were reviewed retrospectively. Full-field ERGs were recorded with dilated pupils before surgery and 41.9 ± 18.3 days afterward using the RETeval system (LKC Technologies, Gaithersburg, MD), with the light-adapted 3.0 and dark-adapted 0.01 stimuli specified by the International Society for Clinical Electrophysiology of Vision (ISCEV) standard. Amplitudes and peak times of the photopic a- and b-waves and the scotopic b-wave were compared using the Wilcoxon signed-rank test, and eyes were grouped according to the presence or absence of CD.

Results

Across the whole cohort, no amplitude or peak time changed significantly. In the CD group, the scotopic b-wave peak time shortened from 120.9 ± 12.0 ms to 114.6 ± 7.8 ms (p = 0.025), while the remaining parameters were unchanged; the non-CD group showed no significant change. Intraocular pressure (IOP) fell from 18.4 ± 4.4 to 8.3 ± 3.5 mmHg after surgery (p < 0.001).

Conclusions

Roughly six weeks after filtration surgery performed alone, ERG amplitudes were unchanged. This is compatible with a limited electrophysiological effect of surgery at this stage or with recovery from changes that occurred earlier, although the design does not allow the two to be distinguished. The shortening of the scotopic b-wave peak time in eyes with CD was an exploratory observation in eight eyes and requires confirmation in larger prospective studies.

## Introduction

In glaucoma, retinal ganglion cells (RGCs) and their axons are lost progressively, and the resulting optic nerve damage is followed by defects in the visual field [1,2]. Reducing intraocular pressure (IOP) is still the only intervention shown to alter this course [2]. As a large proportion of RGCs has already been lost by the time perimetry detects an abnormality [3], objective measures of retinal function are of interest when IOP is lowered surgically. Electroretinography (ERG) offers such a measure: responses obtained under light- and dark-adapted conditions reflect cone- and rod-system function, respectively. One ERG component, the photopic negative response (PhNR), is a slow negative deflection that follows the b-wave and arises largely from RGC activity [4]; its amplitude has been reported to increase once IOP is reduced [5,6]. However, how IOP reduction affects ERG components other than the PhNR has rarely been examined.

Postoperative recordings became practical with the RETeval system (LKC Technologies, Gaithersburg, MD), a handheld device that records ERGs through skin electrodes. Miura et al. used it to follow ERG waveforms across cataract surgery without adverse effects [7,8], and comparable recordings have since been obtained after glaucoma surgery. In eyes free of choroidal detachment (CD), Igawa et al. observed, within a few days of filtration surgery, larger PhNR amplitudes, a higher PhNR/b-wave ratio, and shorter a- and b-wave implicit times, whereas in eyes that developed CD, the amplitudes of the PhNR, a-wave, and b-wave were reduced [9]. To our knowledge, ERG changes approximately six weeks after filtration surgery performed alone have been rarely reported, and dark-adapted responses before and after glaucoma surgery remain uncharacterized.

We therefore used full-field ERG with skin electrodes to describe retinal function approximately six weeks after filtration surgery performed alone. As an exploratory, hypothesis-generating analysis, we also compared cone- and rod-system responses between eyes that did and did not develop CD after surgery.

## Materials and methods

Study design and ethics

Patients who underwent glaucoma filtration surgery at Chiba University Hospital between November 2018 and March 2025 were reviewed retrospectively. The Institutional Review Board of Chiba University Hospital approved the study (approval number: HK202505-02) and, given its retrospective design, waived the requirement for written informed consent; instead, a description of the study was made public, and the patients were given the opportunity to decline participation (opt-out). The study followed the ethical principles set out in the Declaration of Helsinki.

Clinical examinations

Every patient received a full ophthalmic examination that included best-corrected visual acuity (BCVA), IOP measured with a Goldmann applanation tonometer (Haag-Streit, Köniz, Switzerland), slit-lamp biomicroscopy, and indirect ophthalmoscopy. Within the three months preceding surgery, the visual field was examined with the Humphrey Field Analyzer (Carl Zeiss Meditec, Jena, Germany) using the 30-2 Swedish Interactive Threshold Algorithm Standard program. BCVA was obtained monocularly at 3 m with a Japanese standard Landolt C chart (System Charts SC-2000; Nidek Co., Ltd., Gamagori, Japan) and converted to the logarithm of the minimum angle of resolution (logMAR) for analysis.

Inclusion and exclusion criteria

Patients were eligible if filtration surgery had been performed alone for primary open-angle glaucoma, primary angle-closure glaucoma, normal-tension glaucoma, or pseudoexfoliative glaucoma. Eyes with any other disorder capable of reducing visual function and eyes in which cataract surgery had been combined with the glaucoma procedure were not included. As dense lens opacity distorts ERG waveforms, eyes with cataract of Emery-Little grade 3 or above were also excluded [7,8].

Surgical procedures

Three procedures were represented: trabeculectomy, implantation of the Ex-PRESS glaucoma filtration device (Alcon Laboratories, Fort Worth, TX), and implantation of the Preserflo MicroShunt (Santen Pharmaceutical Co., Ltd., Osaka, Japan). No intraoperative complications occurred.

ERG protocol

Before and after surgery, full-field ERGs were obtained with pharmacologically dilated pupils in accordance with the standard of the International Society for Clinical Electrophysiology of Vision (ISCEV) [10], using the "ISCEV 5 step" protocol of the RETeval system. Only two steps of that protocol were recorded: the light-adapted 3.0 ERG (cone response) and the dark-adapted 0.01 ERG (rod response), referred to below as the photopic and scotopic ERGs. Pupils were dilated with topical 0.5% tropicamide and 0.5% phenylephrine hydrochloride. The photopic ERG (3.0 cd·s/m^2^) was recorded after 10 min of light adaptation and the scotopic ERG (0.01 cd·s/m^2^) after 20 min of dark adaptation. No predefined postoperative window was set for the second recording because the study was retrospective; postoperative ERGs were obtained at routine postoperative visits. Figures of the waveforms were prepared with Adobe Illustrator (Adobe Inc., San Jose, CA).

CD classification

Eyes were assigned to the CD group when CD had developed after surgery and had already resolved by the time of the postoperative ERG recording; the remaining eyes formed the non-CD group. Within each group, amplitudes and peak times of the photopic a- and b-waves and the scotopic b-wave were compared before and after surgery. CD was identified on slit-lamp and indirect ophthalmoscopic examination and documented on wide-field fundus images taken with an ultra-widefield scanning laser ophthalmoscope (Optos California; Optos plc, Dunfermline, UK), and its severity was graded with the system described by Shin et al. [11]. Six eyes had grade 1 CD, and two eyes had grade 2 CD, with no more severe grades; CD was first detected at a mean of approximately 15 days after surgery and extended over a maximum of two quadrants. Every affected eye was managed conservatively with observation alone, and none required drainage, anterior chamber reformation, or any other intervention. The date of resolution was not recorded systematically, but resolution was confirmed on examination in every eye before the postoperative ERG was obtained.

Postoperative management

Postoperative management was the same for the three procedures. Every patient received topical 0.1% betamethasone four times daily, tapered over three months, and topical 0.3% gatifloxacin four times daily; no systemic corticosteroids were given. Laser suture lysis was performed when required after trabeculectomy and Ex-PRESS implantation, whereas the Preserflo MicroShunt does not involve scleral flap sutures and therefore did not require suture lysis. No needling or bleb revision was performed in any eye, and no additional treatment was given for postoperative hypotony.

Statistical analysis

Pre- and postoperative amplitudes and peak times of the photopic a- and b-waves and the scotopic b-wave were compared with the Wilcoxon signed-rank test, and pre- and postoperative IOP were compared using the paired t-test. The analyses within the CD and non-CD groups were exploratory; no between-group comparison of the change over time was performed, and no correction for multiple comparisons was applied. A p-value below 0.05 was regarded as significant. All analyses were run in IBM SPSS Statistics for Windows, version 27.0 (IBM Corp., Armonk, NY).

## Results

ERG findings

In the overall cohort, no significant differences were found between the preoperative and postoperative values of any ERG parameter. The mean amplitude and peak time of the photopic a-wave were -6.8 ± 2.3 μV and 13.6 ± 1.7 ms preoperatively and -6.9 ± 3.6 μV and 14.0 ± 1.5 ms postoperatively. The mean amplitude and peak time of the photopic b-wave were 28.3 ± 9.9 μV and 29.8 ± 3.2 ms preoperatively and 26.4 ± 12.9 μV and 29.6 ± 2.2 ms postoperatively. The mean amplitude and peak time of the scotopic b-wave were 41.4 ± 18.1 μV and 109.7 ± 14.1 ms preoperatively and 40.1 ± 19.5 μV and 110.8 ± 15.4 ms postoperatively.

In the CD group, no significant differences in amplitude were observed between the preoperative and postoperative values of any photopic or scotopic ERG parameter. However, the scotopic b-wave peak time shortened significantly after surgery, from 120.9 ± 12.0 ms to 114.6 ± 7.8 ms (p = 0.025). As this group comprised only eight eyes and several parameters were compared without adjustment, this result is an exploratory observation. The photopic a-wave amplitude changed from -5.6 ± 1.4 μV to -5.2 ± 2.5 μV, and its peak time changed from 13.4 ± 2.3 ms to 13.8 ± 1.9 ms. The scotopic b-wave amplitude changed from 40.0 ± 12.0 μV to 39.0 ± 12.8 μV.

In the non-CD group, no significant changes were detected in any ERG parameter. The photopic a-wave amplitude was -7.1 ± 2.4 μV preoperatively and -7.5 ± 3.8 μV postoperatively, with peak times of 13.6 ± 1.6 ms and 14.0 ± 1.4 ms, respectively. The photopic b-wave amplitude and peak time were 28.9 ± 10.9 μV and 29.7 ± 3.4 ms before surgery and 27.8 ± 14.3 μV and 29.5 ± 2.3 ms after surgery. The scotopic b-wave amplitude was 41.8 ± 19.9 μV before surgery and 40.4 ± 21.5 μV after surgery, with peak times of 106.0 ± 12.9 ms and 109.5 ± 17.1 ms, respectively. The preoperative and postoperative ERG parameters of all eyes and the CD and non-CD groups are compared in Table 1.

**Table 1:** Comparison of electroretinography (ERG) parameters before and after surgery Data are presented as mean ± standard deviation. Preoperative and postoperative values were compared using the Wilcoxon signed-rank test. *p < 0.05.

Parameter	All eyes (n = 32)			Non-choroidal detachment (non-CD) group (n = 24)			Choroidal detachment (CD) group (n = 8)		
	Preoperative	Postoperative	p-value	Preoperative	Postoperative	p-value	Preoperative	Postoperative	p-value
Photopic a-wave amplitude (μV)	-6.8 ± 2.3	-6.9 ± 3.6	0.724	-7.1 ± 2.4	-7.5 ± 3.8	0.493	-5.6 ± 1.4	-5.2 ± 2.5	0.446
Photopic b-wave amplitude (μV)	28.3 ± 9.9	26.4 ± 12.9	0.231	28.9 ± 10.9	27.8 ± 14.3	0.689	26.4 ± 6.0	22.0 ± 6.2	0.069
Scotopic b-wave amplitude (μV)	41.4 ± 18.1	40.1 ± 19.5	0.786	41.8 ± 19.9	40.4 ± 21.5	0.864	40.0 ± 12.0	39.0 ± 12.8	1.000
Photopic a-wave peak time (ms)	13.6 ± 1.7	14.0 ± 1.5	0.111	13.6 ± 1.6	14.0 ± 1.4	0.085	13.4 ± 2.3	13.8 ± 1.9	0.672
Photopic b-wave peak time (ms)	29.8 ± 3.2	29.6 ± 2.2	0.776	29.7 ± 3.4	29.5 ± 2.3	0.976	29.9 ± 2.5	29.9 ± 1.7	0.574
Scotopic b-wave peak time (ms)	109.7 ± 14.1	110.8 ± 15.4	0.852	106.0 ± 12.9	109.5 ± 17.1	0.196	120.9 ± 12.0	114.6 ± 7.8	0.025*

Photopic and scotopic ERG waveforms recorded before and after surgery in a representative patient from the CD group are shown in Figure 1, together with wide-field fundus images obtained during CD and after its resolution.

**Figure 1:**
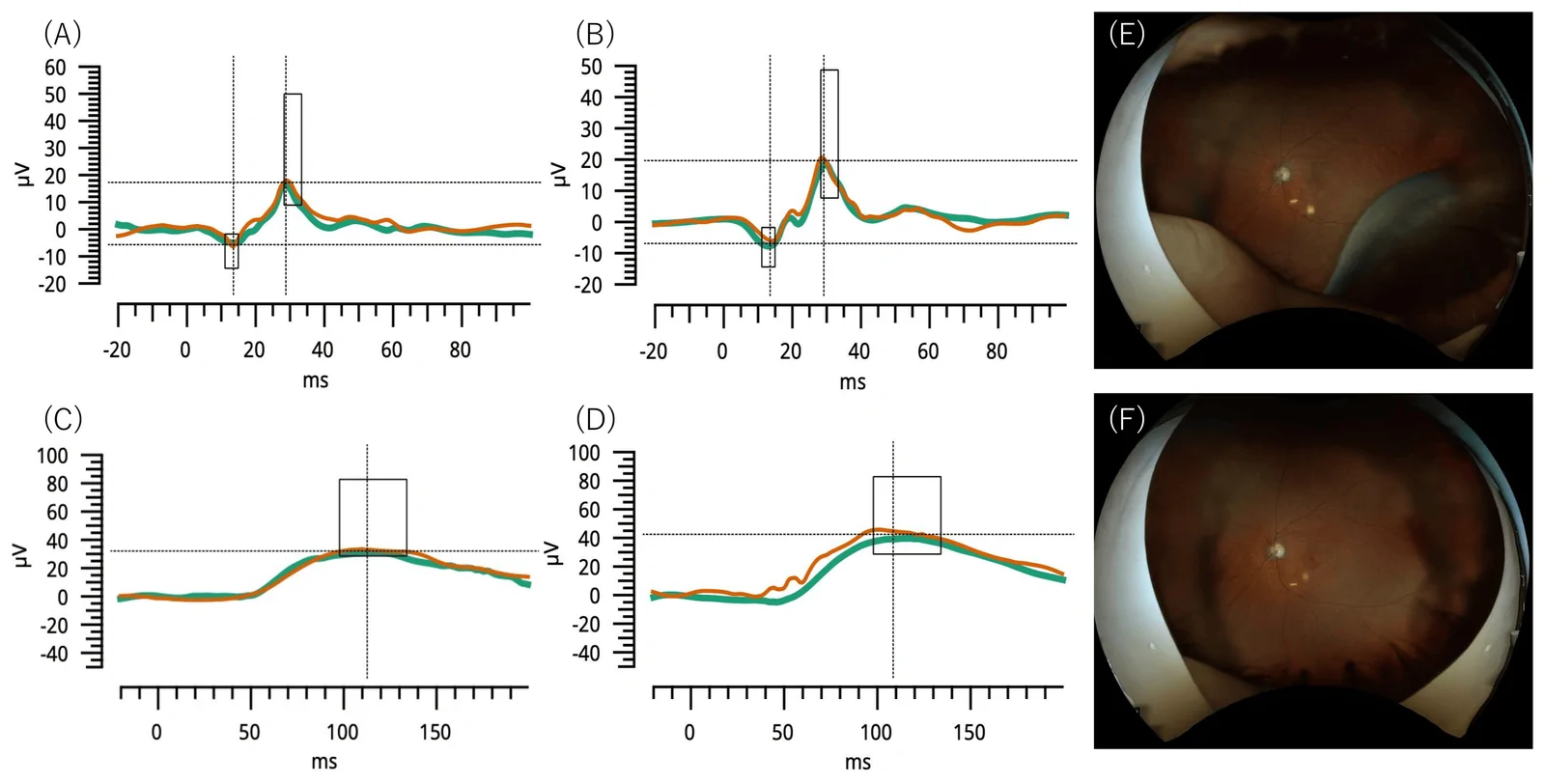
Representative case from the choroidal detachment (CD) group Preoperative and postoperative photopic electroretinography (ERG) waveforms are shown in (A) and (B), respectively, and preoperative and postoperative scotopic ERG waveforms are shown in (C) and (D), respectively. Wide-field fundus images obtained during CD and after its resolution are shown in (E) and (F), respectively. In this patient, intraocular pressure (IOP) was 21 mmHg at the preoperative ERG recording, 8 mmHg during CD, and 10 mmHg at the postoperative ERG recording. No marked change was observed in the photopic ERG, whereas the scotopic ERG showed a slight postoperative shortening of the peak time. In each panel, the two traces represent two recordings obtained under the same conditions (green, first recording; orange, second recording). Dashed lines and boxes are RETeval display elements.

Photopic and scotopic ERG waveforms recorded before and after surgery in a representative patient from the non-CD group are shown in Figure 2.

**Figure 2:**
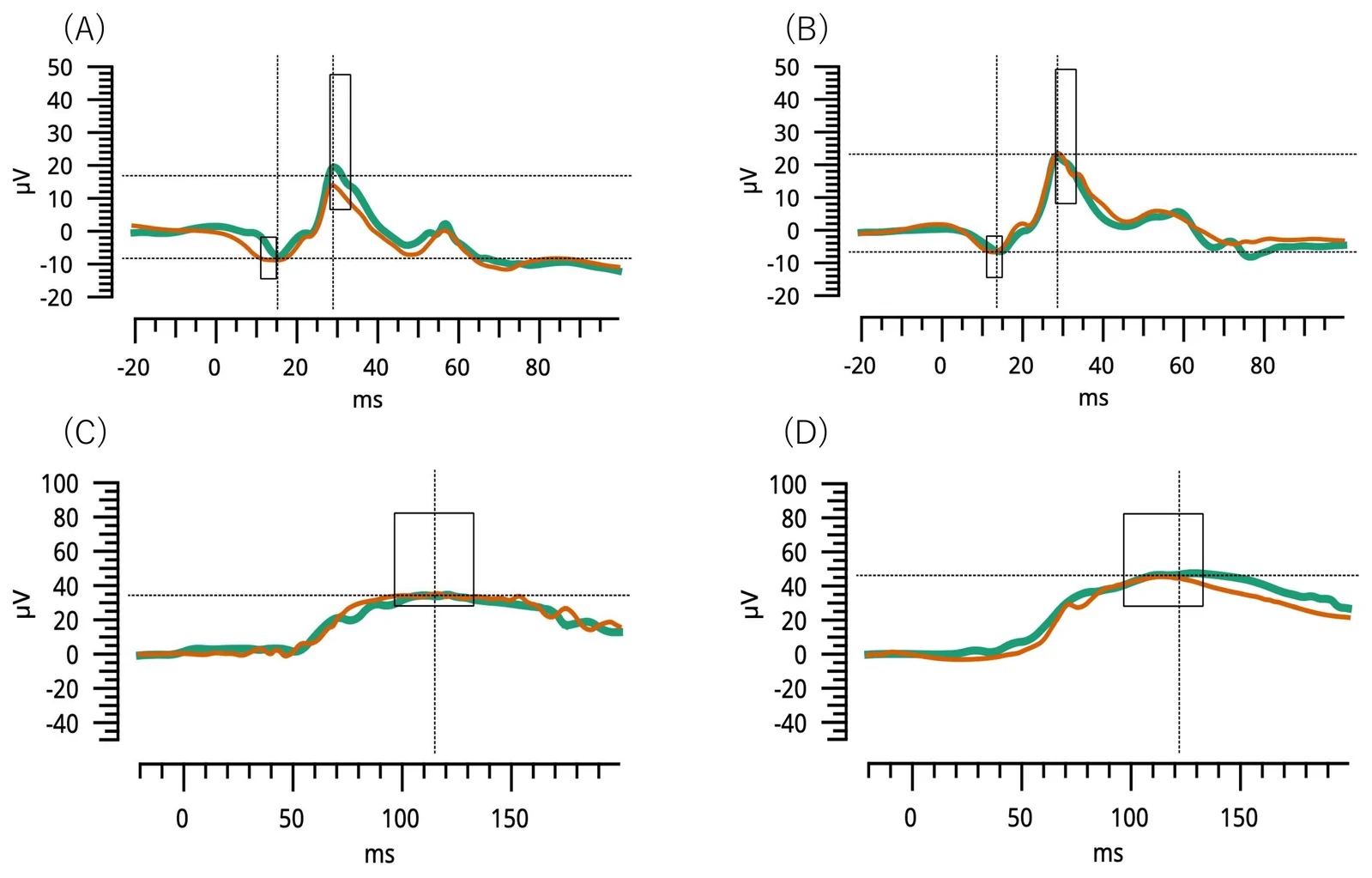
Representative case from the non-choroidal detachment (non-CD) group Preoperative and postoperative photopic electroretinography (ERG) waveforms are shown in (A) and (B), respectively, and preoperative and postoperative scotopic ERG waveforms are shown in (C) and (D), respectively. In this patient, intraocular pressure (IOP) was 20 mmHg at the preoperative ERG recording and 13 mmHg at the postoperative ERG recording. No marked change was observed in either the photopic or scotopic ERG. In each panel, the two traces represent two recordings obtained under the same conditions (green, first recording; orange, second recording). Dashed lines and boxes are RETeval display elements.

## Discussion

Retinal function was assessed electrophysiologically with skin-electrode ERG approximately six weeks after glaucoma filtration surgery. The scotopic b-wave peak time shortened significantly in the CD group, whereas none of the ERG parameters changed significantly in the non-CD group.

Electrophysiological assessment before and after glaucoma filtration surgery has rarely been reported. With skin-electrode ERG, Igawa et al. documented an increase in PhNR amplitude together with shorter a- and b-wave implicit times after surgery [9]. Imaging data point in a similar direction: on optical coherence tomography angiography, the foveal avascular zone becomes smaller after glaucoma surgery, which suggests better capillary perfusion [12]. Lower IOP, improved retinal microcirculation, and functional recovery may therefore be linked, with ERG responses increasing during the early postoperative period.

Lens status also influences these recordings. Miura et al. showed that even mild cataract alters flicker ERGs obtained with the RETeval system: peak times shortened, and amplitudes increased after cataract surgery, and denser cataract was associated with smaller amplitudes [7,8]. They suggested that opacity attenuates the effective stimulus, which lowers amplitudes and delays peak times. Kato et al., also using the RETeval system, described a transient rise in flicker ERG amplitude that peaked one week after cataract surgery [13], implying that postoperative intraocular inflammation, and not only clearing of the media, may enlarge responses. As both studies examined flicker responses, the cellular origin of the amplitude change cannot be specified. Tanikawa et al. similarly found larger scotopic, flicker, and photopic amplitudes one week after cataract surgery, whereas the PhNR was unchanged [14]; they attributed the increase to greater light transmission and altered intraocular light scatter once the opaque lens had been removed. Inflammation after surgery and removal of cataract-related light scatter may thus both enlarge ERG responses.

The present cohort differed in two respects: cataract surgery was never combined with the filtration procedure, and ERGs were obtained roughly six weeks postoperatively (41.9 ± 18.3 days). Combined surgery, surgical trauma, and inflammation all influence ERG waveforms, so this design was intended to limit such confounders and to isolate the effects of IOP reduction and CD on retinal function.

A shortening of the scotopic b-wave peak time was seen in the CD group, although this exploratory observation rests on eight eyes and requires confirmation. Concerning the effect of CD on retinal function, Igawa et al. reported smaller a-wave, b-wave, and PhNR amplitudes in eyes with postoperative CD [9]. Miyake et al. noted subnormal preoperative electrooculogram values in eyes with rhegmatogenous retinal detachment accompanied by CD, which suggests that impaired choroidal circulation can compromise the outer retina [15]. In a separate report, the same group found that retinal function declined transiently while subretinal fluid was drained and returned as IOP increased, indicating sensitivity to transient hypotony [16]. Neither study examined rod-driven responses, but the change we observed in the scotopic peak time may likewise reflect recovery of choroidal or outer retinal function once CD had resolved.

The components analyzed here do not address the cells that are central to glaucoma. The a-wave originates mainly from photoreceptors, the b-wave mainly from bipolar cells, and the dark-adapted 0.01 response reflects rod-driven activity, whereas the PhNR arises largely from RGCs. The stability of these components at approximately six weeks therefore indicates that outer and middle retinal function was unchanged at this time point but provides evidence neither for nor against recovery of RGC function after IOP reduction. This also explains why the present findings are compatible with the early improvement in PhNR amplitude reported after filtration surgery [9]. A valid PhNR measurement at this time point would have shown directly whether such an RGC-level improvement persisted into the mid-postoperative period or was transient and, in eyes with CD, whether the reduction in PhNR amplitude described after surgery recovered once the detachment resolved. Addressing these questions requires the ISCEV extended protocol for the PhNR, with a red flash on a rod-saturating blue background [4], rather than the light-adapted 3.0 stimulus used here.

No ERG amplitude changed significantly in our cohort. The relatively late timing of the postoperative recordings and the exclusion of eyes with combined cataract surgery may account for this. The findings are compatible with recovery, by roughly six weeks, of the electrophysiological dysfunction that accompanies CD in the early postoperative period, but they do not demonstrate such a trajectory: the same eyes were not examined serially during the early postoperative period, and only preoperative and later postoperative recordings were compared. The small cohort limits generalizability, and serial recordings at fixed postoperative time points are needed.

Several limitations apply, of which those concerning the statistical design are the most important. The cohort comprised 32 eyes, with only eight eyes in the CD group, so the analyses were underpowered; the absence of significant amplitude changes cannot be taken as evidence that no change occurred, and a type II error cannot be excluded. The single significant result rests on those eight eyes and on several unadjusted comparisons and would not survive Bonferroni correction, so the subgroup analysis is exploratory and hypothesis-generating rather than confirmatory. No between-group comparison of the change over time was performed; the within-group tests used here cannot establish whether the magnitude of change differed between eyes with and without CD, and a prospective study powered for such a comparison, with adjustment for the baseline value, is required. The CD group also had a longer preoperative scotopic b-wave peak time than the non-CD group, so regression toward the mean cannot be excluded; the postoperative value in the CD group (114.6 ± 7.8 ms) nevertheless remained longer than the preoperative value in the non-CD group (106.0 ± 12.9 ms), so the change is not explained by convergence toward the values of the other group, and the two possibilities cannot be separated in a subgroup of this size. The small cohort follows from the restrictive inclusion criteria because eyes with simultaneous cataract surgery and eyes with cataract of Emery-Little grade 3 or above were excluded to avoid confounding of the ERG waveforms.

The design was also retrospective: the interval between surgery and the postoperative recording varied from 14 to 85 days because no predefined window was set, which may obscure temporal patterns, and the date of CD resolution was not recorded systematically, although resolution was confirmed in every eye before the recording. Three procedures with potentially different profiles of hypotony, inflammation, and CD were combined; the anti-inflammatory regimen was identical for all of them, but the group sizes did not permit a procedure-stratified analysis. ERG data from the early postoperative period and long-term follow-up were unavailable. Correlations between ERG parameters and clinical measures such as IOP and visual field indices were not examined: visual fields were obtained only once, within three months before surgery, and postoperative visual field data were not available, so changes in ERG parameters could not be related to changes in visual field parameters. Finally, the PhNR, which arises largely from RGC activity and is the ERG component most specifically related to glaucomatous damage, had to be excluded because the values extracted from the waveforms could not be considered reliable; the stimulus used here was also not the red flash on a rod-saturating blue background recommended for PhNR recording [4]. The influence of IOP reduction and CD on RGC function therefore remains unknown, and a prospective study combining a PhNR-specific protocol with visual field testing at matched time points is required. Grouping by the presence or absence of CD also left the relationship between CD severity and ERG change unexamined; Igawa et al. suggested that PhNR reduction may relate to CD severity [9], making severity-stratified analysis a further target for future work.

## Conclusions

Approximately six weeks after glaucoma filtration surgery performed alone, ERG amplitudes were essentially unchanged. The only significant postoperative difference in peak time was a shortening of the scotopic b-wave in eyes that had developed CD. Because this exploratory finding is based on eight eyes and several unadjusted comparisons, and the preoperative value in this group was longer than that in the non-CD group, it requires confirmation. The results are compatible with, but do not demonstrate, resolution over time of ERG alterations occurring soon after filtration surgery, since the same eyes were not examined serially during the early postoperative period. Larger prospective series with recordings at fixed time points spanning both the early and the later postoperative period, and including the photopic negative response, are required for a more accurate picture of retinal function after surgery.
